# Visual and corresponding tactile dataset of flexible material for robots and cross modal perception

**DOI:** 10.1016/j.dib.2024.110836

**Published:** 2024-08-13

**Authors:** Shuchang Xu, Haohao Xu, Fangtao Mao, Menghui Ji, Wenzhen Yang

**Affiliations:** a School of Information Science and Technology, Hangzhou Normal University, Hangzhou, Zhejiang 311121, China; b Research Center for Humanoid Sensing, Intelligent Perception Research Institute of Zhejiang Lab, Hangzhou, Zhejiang 311121, China

**Keywords:** Tactile sensor, Visual-tactile fusion, Defect detection, Cross modal perception, Cross modal generation, Leather defect, Image segmentation, Flexible material

## Abstract

Humans primarily understand the world around them through visual perception and touch. As a result, visual and tactile information play crucial roles in the interaction between humans and their environment. In order to establish a correlation between what is seen and what is felt on the same object, particularly on flexible objects (such as textile, leather, skin etc.) which humans often access by touch to cooperatively determine their quality, the need for a new dataset that includes both visual and tactile information arises. This has motivated us to create a dataset that combines visual images and corresponding tactile data to explore the potential of cross-modal data fusion. We have chosen leather as our object of focus due to its widespread usage in everyday life. The dataset we propose consists of visual images depicting leather in various colours and displaying defects, alongside corresponding tactile data collected from the same region of the leather. Notably, the tactile data comprises components along the X, Y, and Z axes. To effectively demonstrate the relationship between visual and tactile data on the same object region, the tactile data is aligned with the visual data and visualized through interpolation. Considering the potential applications in computer vision, we have manually labelled the defect regions in each visual-tactile sample. Ultimately, the dataset comprises a total of 687 records. Each sample includes visual images, image representations of the tactile data (referred to as tactile images for simplicity), and segmentation images highlighting the defect regions, all with the same resolution.

Specifications Table

**Table:** 

Subject	Computer Vision and Pattern Recognition, Human-Computer Interaction, Computer Science Applications, Multimedia
Specific subject area	Defect detection, Vision-Tactile fusion, Vision-Tactile data generation
Data format	Raw
Type of data	Image
Data collection	We have chosen Crazy Horse Leather (CHL) as the primary flexible material for our study. In order to collect visual and tactile data, we have designed a specific platform capable of simultaneously gathering cross-modal data. The platform consists mainly of a three-axis linkage controlled by servo motors, a tactile data collection module, and a digital camera with an illuminator. Visual images are captured by the digital colour IP-camera with a resolution of 6 million pixels. Tactile data is captured by a tactile three-axis force sensor with a measurement range of 0-2N and an accuracy of approximately 0.5 %. The force sensor is mounted on the Z-axis of the linkage and can be moved along the X- or Y-axes. The tactile signal, consisting of XYZ components, is collected in XY space line by line as the X- or Y-axis moves. All tactile signals are then sampled by the FPGA module. To align with visual data, the tactile data is interpolated and visualized to maintain the same resolution as the visual image. Finally, we select visual images and tactile images of the X- and Z-axes to form our proposed dataset. For more information about the data acquisition setup, please refer to section 3.
Data source location	***Institution****:* Intelligent Perception Research Institute of Zhejiang Lab ***City/Town/Region****:* Hangzhou, Zhejiang Province ***Country****:* China
Data accessibility	Repository name: CHL_Visual_Tactile_Dataset Data identification number: 10.17632/j7pz7x4wmb.4 Direct URL to data: https://data.mendeley.com/datasets/j7pz7x4wmb/4
Related research article	Haohao Xu, Shuchang Xu, Wenzhen Yang. Unsupervised industrial anomaly detection with diffusion models, Journal of Visual Communication and Image Representation, Volume 97, 2023. https://doi.org/10.1016/j.jvcir.2023.103983.

## 1 Value of the Data

The dataset is valuable in the research fields of humanoid robots, defect detection, and visual-tactile data generation.

• Humanoid robots typically rely on visual and tactile perception to interact with the environment [[1], [2], [3]]. Various public datasets have been introduced that contain visual and tactile data acquired from clothing, household items, and fabric, such as ViTac [4], PHAC-2 [5], Grasp dataset [6], and GelFabric [7] et al. However, these existing datasets collect the visual and tactile data from different objects. The proposed dataset focuses on visual and tactile data acquired from the same region of the same object and is expected to be useful for the robot function of “touching to see”.
• Visual inspection and grading systems are widely used in the industry to ensure high product quality. A few defect datasets, such as KolektorSDD [8] and NEU-DET [9], have been released to train neural networks to detect defects, most of which are surface defects. This dataset includes both surface defects and underside defects, which can only be noticed in tactile data. Additionally, the tactile data along the X axes provide extra information on the roughness of the object's surface, which is highly useful for product quality grading.
• Cross-modal data generation is currently a hot topic in artificial intelligence, with data being the dominant element in this field. Estimating tactile properties from vision or visual properties from tactile [10,11] requires visual data and corresponding tactile data. Unlike existing researches that use visual and sparse tactile data based on GelSight, the proposed dataset provides visual and corresponding dense tactile data, leading to more accurate cross-modal generation.
• Researchers can also use this dataset as a benchmark to evaluate the performance of algorithms in areas such as defect detection, visual-tactile data generation, and leather quality assessment.

## 2 Background

Quality control of flexible materials requires consideration of defects on both of surface and subsurface. Overall quality measurement cannot depend solely on vision-based methods. Taking leather as an example, material quality may ultimately be determined by human touch. This motivates us to collect both of visual and tactile data to comprehensively evaluate product quality. On the other hand, cross-modal fusion, as well as datasets that include visual and tactile data, has become increasingly popular in research fields related to humanoid robotics in recent years, as humans interact with the environment through both seeing and touching. However, there are few datasets that include both visual and corresponding tactile data from the same region of the same object. Therefore, we have decided to build a cross-modal dataset that includes both visual data and corresponding tactile data from the same region. We hope that researchers in areas such as defect detection, cross-modal data fusion and generation, and human-computer interaction will benefit from our proposed dataset.

## 3 Data Description

**Fig. 1:**
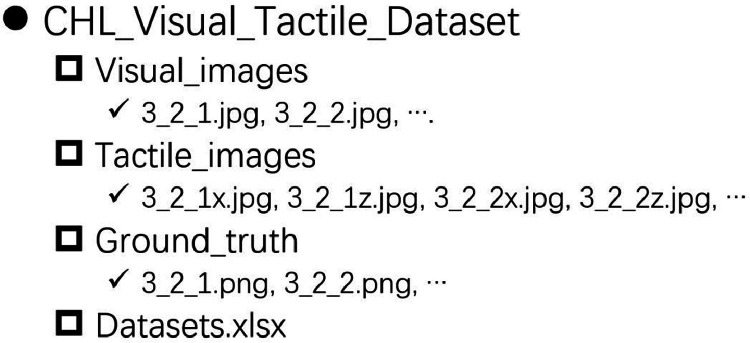
The folder organization structure of the visual and tactile images.

The dataset contains raw visual images, image representations of the tactile data along the X- and Z-axes and an Excel file that organize every sample and their correspondence in order. The tactile images are interpolated on the raw haptic signal to align with the visual images. Both the visual and tactile images have identical resolution of 620×410. All the data are organized using the folder structure described in Fig. 1 As we can see, the dataset consists of a sheet along with three types of data: visual images, tactile images, and segmentation images. All data is represented as images and stored in separate subfolders.

**Fig. 2:**
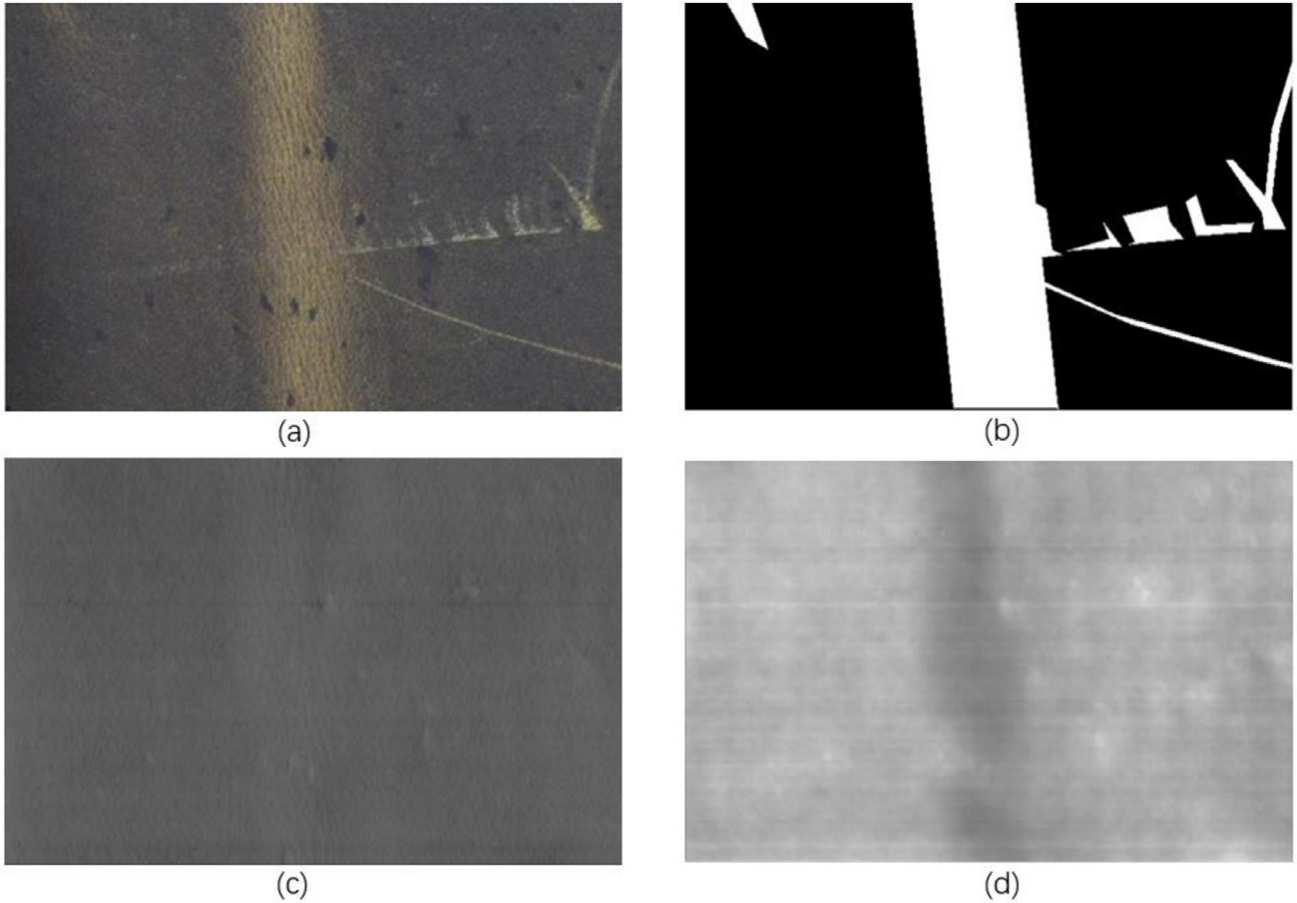
Each record consists of one visual image (a), two tactile images along the X (c) and Z (d) axis, and one segmentation image(b), which marks defect regions of the leather.

The dataset consists of 743 records. Each record includes one visual image, two tactile images along the X and Z axes, and one defect segmentation image, as shown in Fig. 2. Tactile image filenames ending with x and z denote X and Z components respectively.

**Fig. 3:**
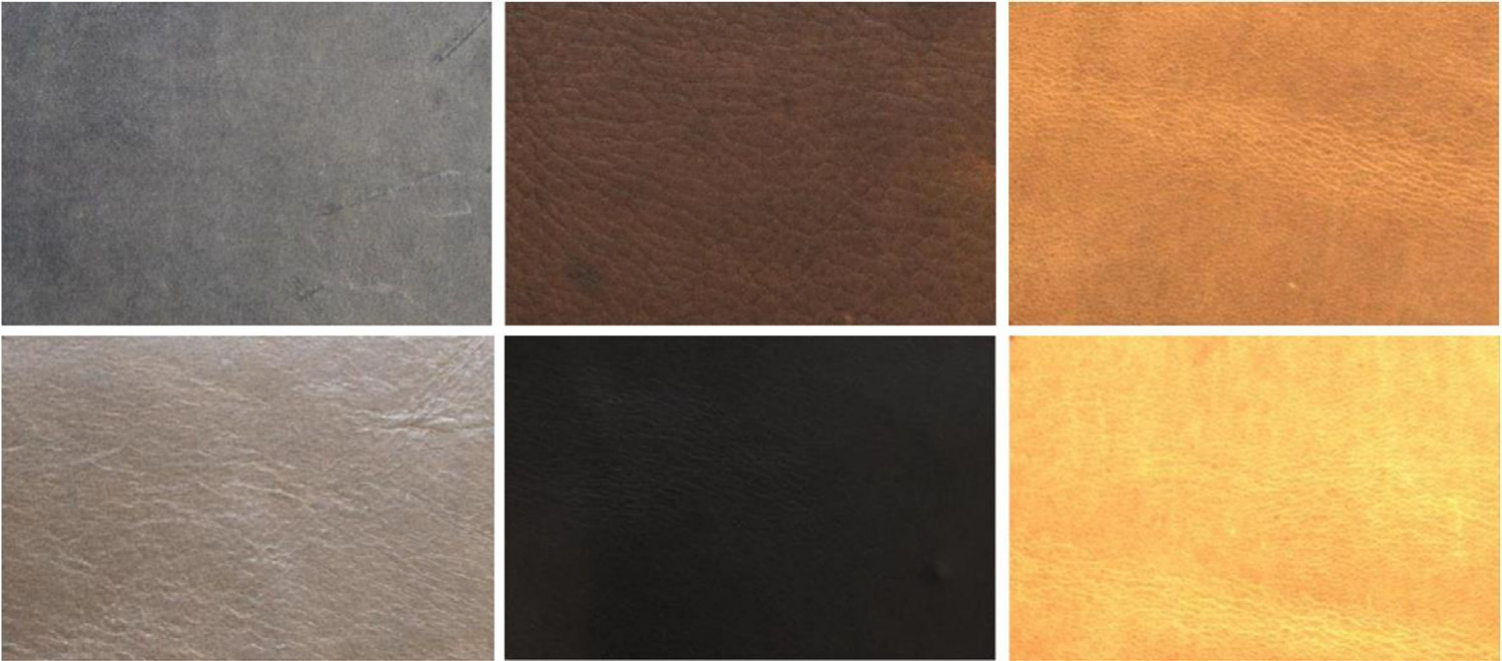
Visual image samples in different surface colors.

**Fig. 4:**
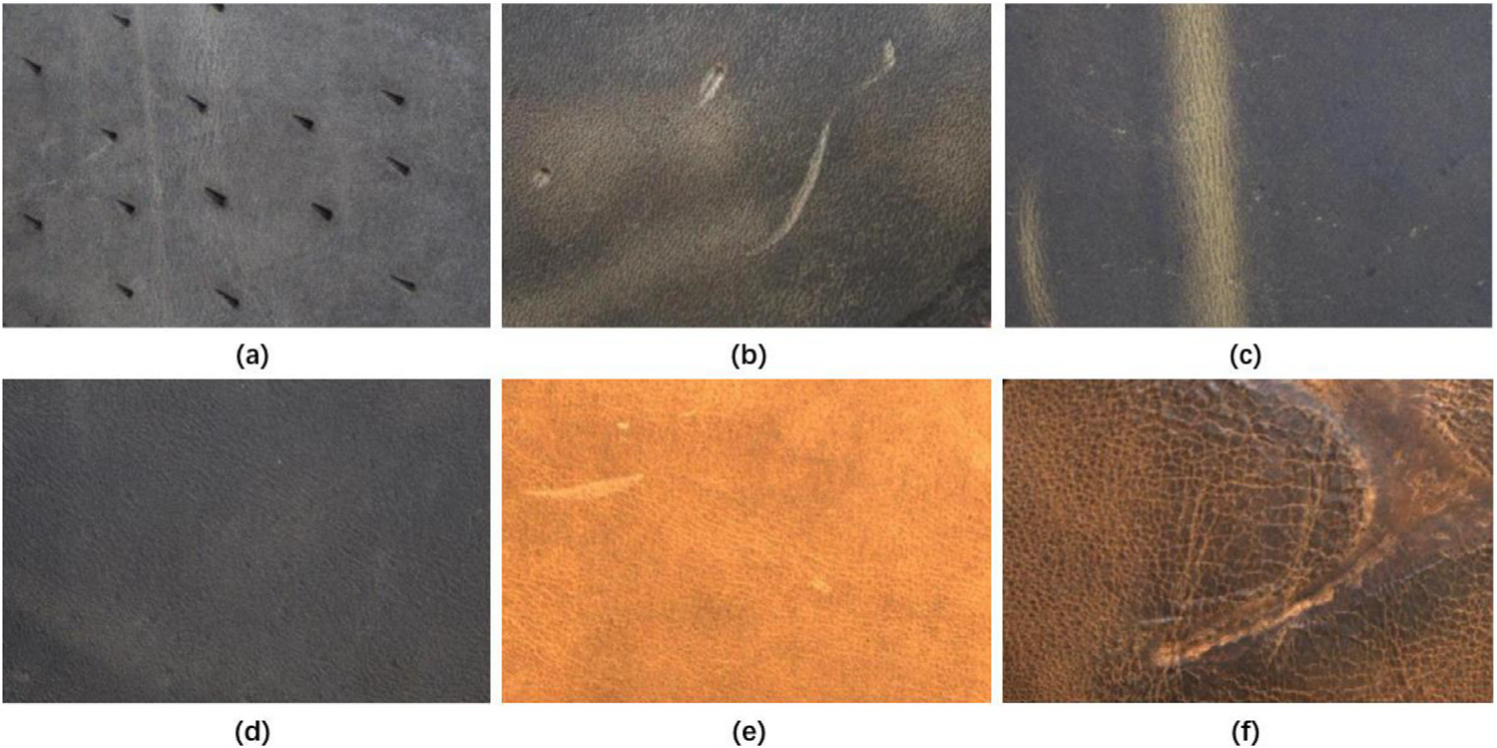
Visual images in different defect textures and severities. Leather defects may have dotted (a), linear (b) (e), striped (c) and blocky (f) shapes. All leather images show noticeable surface defects with different severities except for image (d), which has no defect.

The samples in the dataset exhibit a wide range of colors and textures. Fig. 3 illustrates visual images with different colors of leather, while Fig. 4 shows visual images with defects of varying shapes and distributions.

**Fig. 5:**
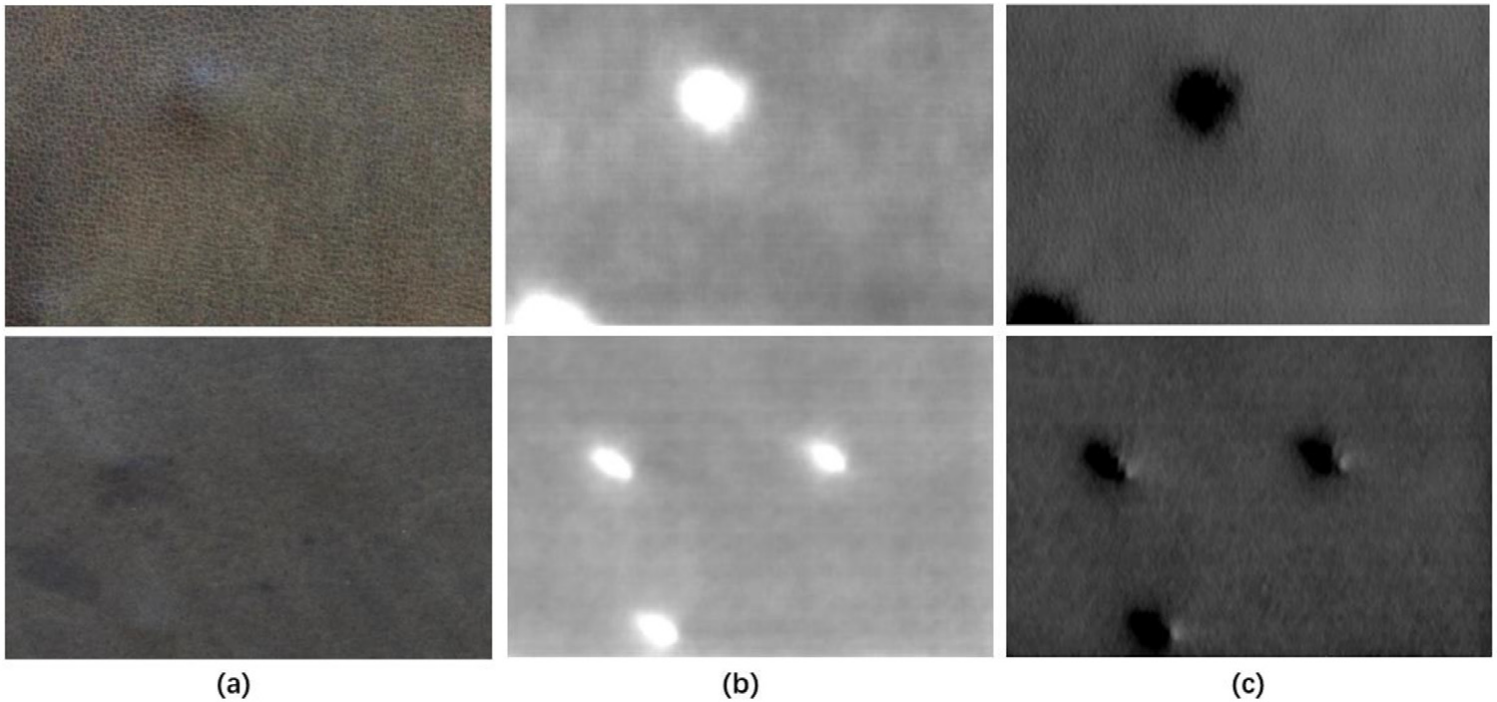
Underside defects which are not noticeable in visual images (a) but are quite noticeable in tactile image both along Z axis (b) and X axis (c).

Moreover, the dataset demonstrates the advantage of cross-modal data fusion. As a flexible material, leather may have defects on its surface and underside, which can be observed in the visual and tactile images, respectively. Combining visual and tactile images provides better information on the distribution of defects. Fig. 5 showcases underside defects that are not noticeable in visual images but are noticeable in tactile images along both the Z-axis (b) and X-axis.

## 4 Experimental Design, Materials and Methods

**Fig. 6:**
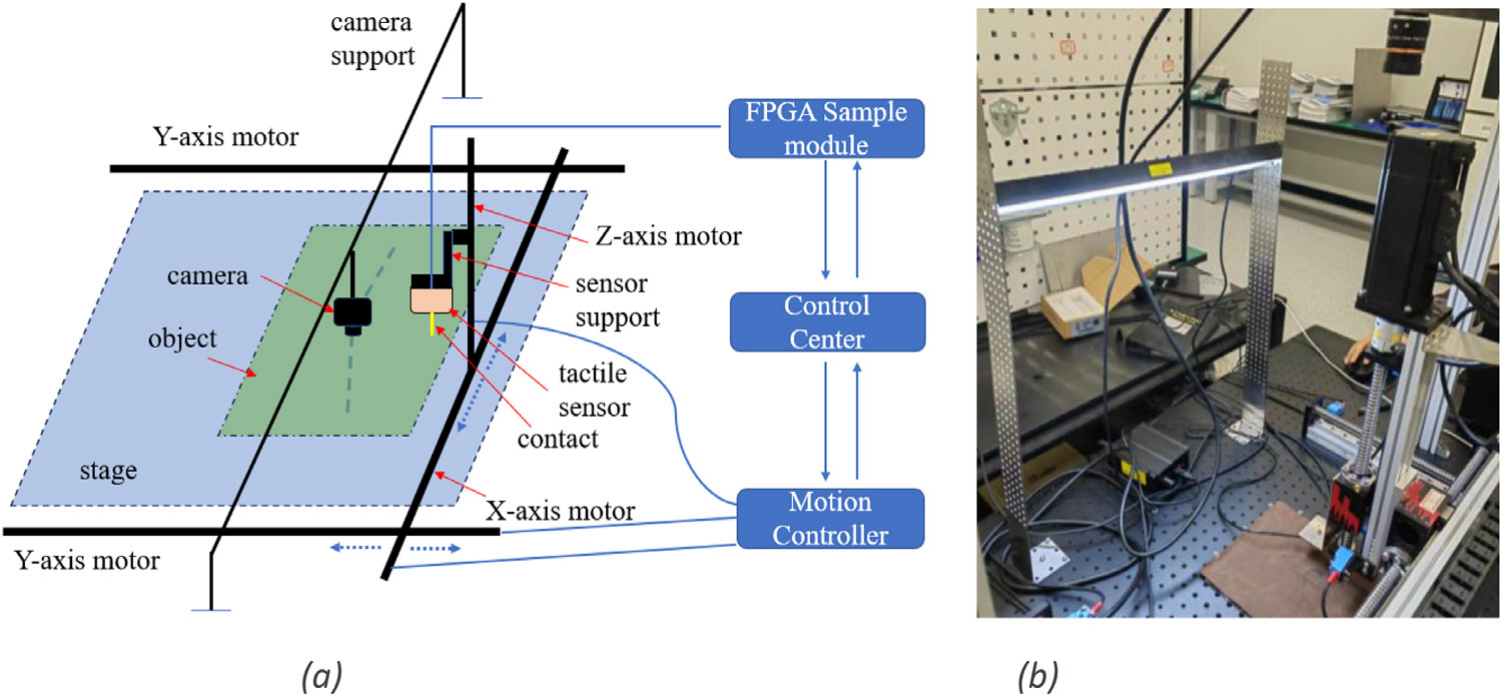
We build a specific platform to capture both of visual and tactile data. (a) The equipements layout of data collection environment. (b) The snapshot of data collection platform.

We have selected Crazy Horse Leather (CHL) as a representative leather material due to its widespread use and dominant position in the high-end cowhide market. In order to simultaneously capture visual and tactile data, we have designed a dedicated platform, as illustrated in Fig. 6. The platform primarily consists of a three-axis linkage, a digital camera, and a tactile data sampling module for acquiring visual and tactile data, respectively.

**Fig. 7:**
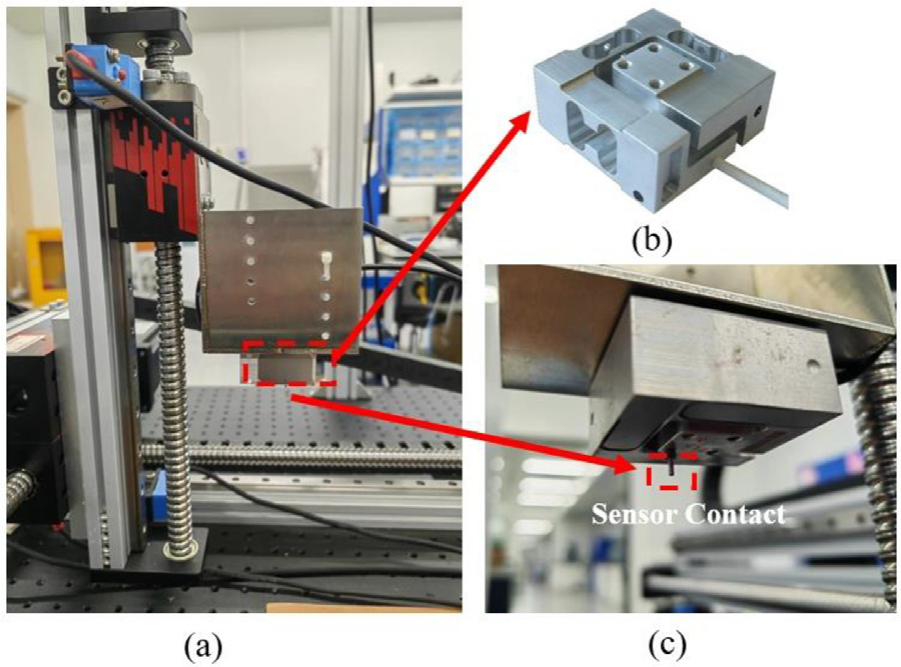
The three-axis force sensor used to collect tactile data. (a) A L-shape bracket is designed to mount the force sensor. (b) Image of force sensor. (c) The contact tip of the force sensor faces the object stage.

The three-axis force sensor (Fig. 7b, Type:K3D40), featuring a spherical contact tip (Fig. 7c) with a radius of 0.5mm, is mounted on the Z-axis by a L-shape bracket (Fig. 7a). The sensor can then be moved within the plane space along the X- or Y-axis. Dense tactile data of the leather is collected as the force sensor is carried and moved along the X- and Y-axes by servo motors, which are controlled by a motion controller as depicted in Fig. 6. The force sensor has a measurement range of 0-2N and an accuracy of approximately 0.5 %.

**Fig. 8:**
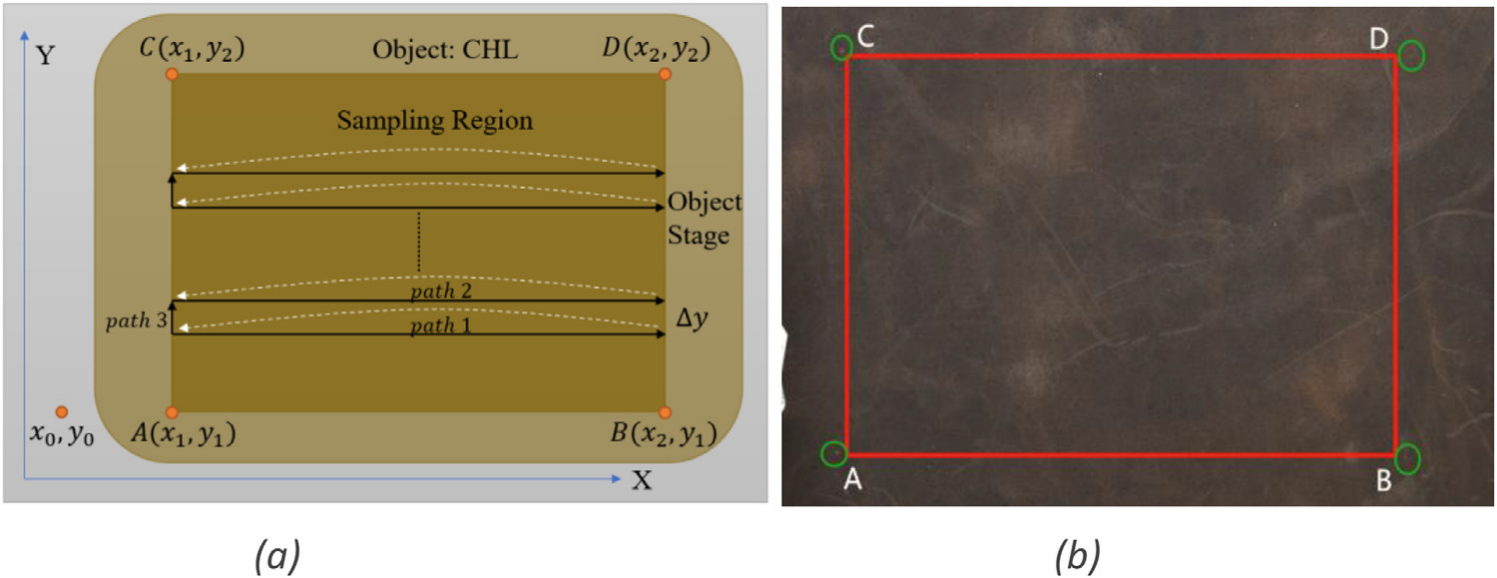
Dense tactile data collection and visual-tactile data alignment. (a) Strategy illustration of tactile data collection. (b) The region of tactile data is defined by four marks left by the contact tip of the force sensor. We apply red ink in contact tip before tactile data collection processing. Zoom in to clearly see the marks.
(2) Collecting dense tactile data line by line. The tactile sensor is then moved to acquire tactile data along path1, as shown in Fig. 8(a). Every 5ms, the axis servo motor and tactile sensor continuously provide the corresponding coordinates and force values, respectively, after sampling by the FPGA module. Specifically, the force value contains XYZ three components. Upon reaching point B, the sensor will return to point A and move up △y along path3, as shown in Fig. 8a. Subsequently, a new round of line tactile data collection (see path2 in Fig.8a) repeats until the data collection is completed within the region of ABCD, as illustrated in Fig. 8(a).
(3) Aligning the visual data. Typically, the range of visual data acquisition is much larger than that of tactile data. Therefore, we clip the visual image to maintain the same data region according to the four corner marks left by the contact tip of the tactile sensor, as shown in Fig. 8(b).
(4) Tactile data interpolation. The tactile data is initially sampled by the FPGA module and stored in the control center. Subsequently, we apply cubic spline interpolation on the tactile data, followed by data visualization, to maintain the same resolution as the visual image.

The visual data is captured straightforwardly by a digital IP-camera. As shown in Fig. 6(a), the camera is fixed on the support of the stage. It is located to fully capture the image of the entire object on the stage without the need for any movement. However, it is necessary to move the force sensor along the Z-axis to approach the object and along the X- and Y-axes to collect tactile data. The whole process of collecting tactile data is relatively complex and time-consuming. Initially, we apply red ink to the contact tip of the tactile sensor to mark the tactile data region and facilitate subsequent alignment of visual-tactile data. The tactile data collection proceeds as follows:

(1) Moving to the starting point. The tactile sensor will descend until it makes contact with the object surface while the Z-axis moves down. The contact point is then marked as A(x1, y1), as depicted in Fig. 8.

Considering the sampling distance along the Y direction (as shown in Fig. 8a), the tactile data value of the y component is relatively unsignificant. Therefore, the proposed dataset only includes tactile components of the X and Z axes.

## Limitations

Acquiring dense tactile data for flexible objects is a time-consuming process. In particular, collecting tactile signals from a leather sample measuring 10 cm * 15 cm typically requires 5–6 h. As a result, it took us several months to create the dataset we propose. Additionally, we mark the tactile region using red ink on the tactile sensor contact. However, the red ink will fade after several contacts, resulting in a blurry mark. Therefore, we often check the mark to ensure its visibility. Moreover, the size of the tactile sensor contact also impacts the size of the mark, which further affects the alignment accuracy between the tactile and visual data.

## Ethics Statement

The authors have read and follow the ethical requirements for publication in Data in Brief. We confirmed that no human subjects were involved in this study. Therefore, informed consent and ethical committee approval were not required. Animal experiments were not conducted. Data collected from social media platforms was not used in this research. Therefore, participant consent and data redistribution policies were not applicable.

## CRediT authorship contribution statement

**Shuchang Xu:** Writing – original draft, Software. **Haohao Xu:** Writing – review & editing, Data curation, Software, Validation. **Fangtao Mao:** Data curation, Visualization, Methodology. **Menghui Ji:** Software, Methodology. **Wenzhen Yang:** Supervision, Resources, Funding acquisition.

## Data Availability

CHL_Visual_Tactile_Dataset (Original data) (Mendeley Data).
